# The genome sequence of the Common Moorhen, *Gallinula chloropus* (Linnaeus, 1758) (Gruiformes: Rallidae)

**DOI:** 10.12688/wellcomeopenres.24858.1

**Published:** 2025-10-03

**Authors:** Toby D. Humby, Michelle F. O’Brien, Rosa Lopez Colom, Natural History Museum Genome Acquisition Lab, Wellcome Sanger Institute Tree of Life Management, Samples and Laboratory team, Wellcome Sanger Institute Scientific Operations: Sequencing Operations, Wellcome Sanger Institute Tree of Life Core Informatics team, Tree of Life Core Informatics collective, Darwin Tree of Life Consortium

**Affiliations:** 1 Wildfowl & Wetlands Trust, Slimbridge, Gloucestershire, England, UK

**Keywords:** Gallinula chloropus; Common Moorhen; genome sequence; chromosomal; Gruiformes

## Abstract

We present a genome assembly from an individual male *Gallinula chloropus* (Common Moorhen; Chordata; Aves; Gruiformes; Rallidae). The assembly contains two haplotypes with total lengths of 1 282.39 megabases and 1 208.56 megabases. Most of haplotype 1 (92.66%) is scaffolded into 38 chromosomal pseudomolecules, including the Z sex chromosome. Haplotype 2 was assembled to scaffold level. The mitochondrial genome has also been assembled, with a length of 17.04 kilobases. This assembly was generated as part of the Darwin Tree of Life project, which produces reference genomes for eukaryotic species found in Britain and Ireland.

## Species taxonomy

Eukaryota; Opisthokonta; Metazoa; Eumetazoa; Bilateria; Deuterostomia; Chordata; Craniata; Vertebrata; Gnathostomata; Teleostomi; Euteleostomi; Sarcopterygii; Dipnotetrapodomorpha; Tetrapoda; Amniota; Sauropsida; Sauria; Archelosauria; Archosauria; Dinosauria; Saurischia; Theropoda; Coelurosauria; Aves; Neognathae; Neoaves; Gruiformes; Rallidae; *Gallinula*; *Gallinula chloropus* (Linnaeus, 1758) (NCBI:txid9123)

## Background

The common moorhen ( *Gallinula chloropus*) is a small waterbird in the rail family. Adult birds are black all over with stark white rumps and a white horizontal line marking on their flanks. The white rump is easily spotted, as the bird habitually flicks its tail upwards to reveal it while moving, and also displays it during territorial disputes. Their yellow legs with long, unwebbed toes and red facial shield provide striking contrast to their monochrome plumage. The facial shield extends from above the red eyes onto the short, pointed bill, which ends in a yellow tip. Juveniles do not show a red shield until maturity and take on a duller grey plumage and leg colour until adulthood ( del Hoyo J *et al.*, 1996). When flushed, individuals prefer to run or swim rather than fly, though they are capable of low, short flights between dense cover. Despite this tendency to short, hesitant flight, some northern populations can migrate distances to escape freezing winter conditions that would otherwise limit their aquatic habits ( del Hoyo J *et al.*, 1996).

Moorhens are found across the Old World, with resident and breeding populations found throughout mainland Europe and northern Africa across to southern Africa and south-east Asia ( eBird, 2025). In 2011, the species was split from the now recognised common gallinule ( *Gallinula galeata*), a similar-looking New World species. The common moorhen itself now has five recognised geographically separated subspecies ( Gill *et al.*, 2025).

Moorhens are wetland specialists of marshes and reedbeds which are used for cover and nesting material. They make small bowls of reeds with 3 to 15 eggs and both parents of seasonally monogamous pairs taking an active role in rearing, although intraspecific brood parasitism is not uncommon ( Amininasab *et al.*, 2021; Foreman, 2001; Huxley & Wood, 1976; Petrie, 1987). Moorhens have a wide diet mostly consisting of vegetation such as grasses and aquatic invertebrates such as *Daphnia* ssp. ( Lardjane-Hamiti *et al.*, 2015), but also feed opportunistically on fruits and nuts ( Thomas, 1982) and even carrion ( Ciach, 2004).

The species is shot recreationally during hunting seasons ( BASC, 2025). The IUCN ranks the common moorhen as least concern, due to the species’ wide distribution and stable population ( IUCN, 2019). As moorhen are abundant and widespread, present in high densities in many wetlands, some studies have used moorhens as subjects to examine heavy metal and metalloid build-up in bird populations in polluted environments ( López-Perea *et al.*, 2019; Zamani-Ahmadmahmoodi *et al.*, 2009).

Being a wetland specialist and ground feeder, the moorhen is vulnerable to avian botulism, contracted via invertebrate prey and also through sediment disturbance. It is also infected with avian influenza through contact with high-densities of flocking birds ( Anza *et al.*, 2016; Martelli *et al.*, 2025). Analysis of the genetic sequence of this species could be applied to epidemiological studies looking at immune response and disease spread.

**Figure 1.**
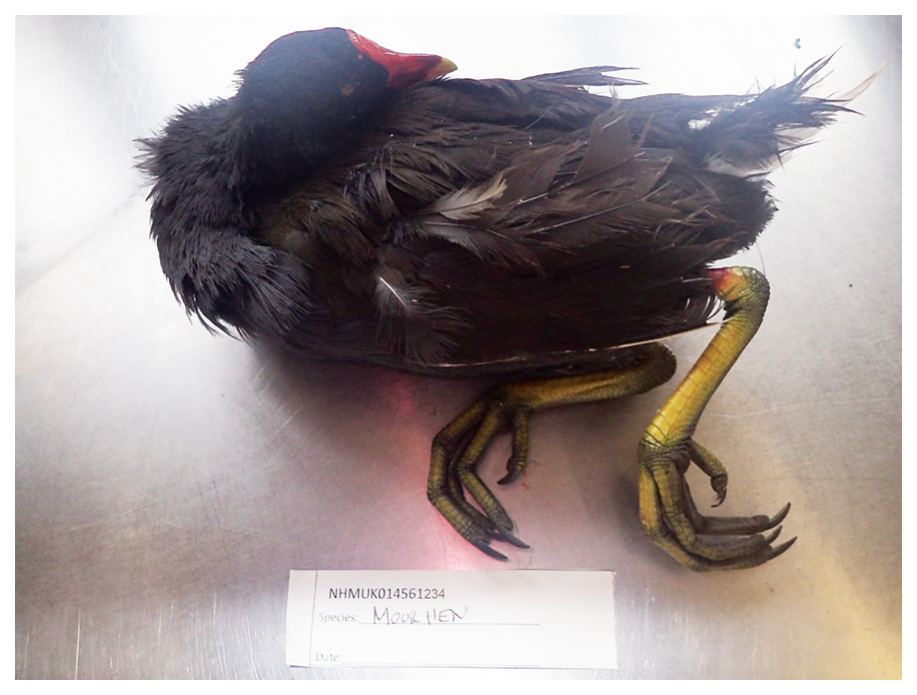
Photograph of the *Gallinula chloropus* (bGalChl1) specimen used for genome sequencing.

Genetic analysis of common moorhens in the literature has focused on population dynamics such as dispersal patterns ( Ruan *et al.*, 2018), genetic diversity ( Ruan *et al.*, 2018; Van Duyse *et al.*, 1999), and population viability of endangered subspecies ( Miller *et al.*, 2015). We present a chromosomally complete genome sequence for *Gallinula chloropus*, produced using the Tree of Life pipeline from a specimen collected in London Wetlands Centre, London, England, United Kingdom ( Figure 1), as part of the Darwin Tree of Life project.

## Methods

### Sample acquisition

The *Gallinula chloropus* specimen used for genome sequencing (specimen ID NHMUK014561234, ToLID bGalChl1; Figure 1) was a wild male specimen found deceased and collected at WWT London Wetland Centre, London in 2021 as part of a disease surveillance programme carried out by WWT in contribution to the Great Britain Wildlife Health Partnership. It was collected from London Wetlands Centre, London, England, United Kingdom (latitude 51.47, longitude –0.23) on 2021-08-14 and stored at –20 °C. Several small samples of pectoral muscle were taken and stored at –80 °C. The specimen was collected and identified by Michelle O’Brien (Wildfowl & Wetlands Trust). A sample from the same specimen was used for RNA sequencing. Sample metadata were collected in line with the Darwin Tree of Life project standards described by Lawniczak *et al*. (2022).

### Nucleic acid extraction

Protocols for high molecular weight (HMW) DNA extraction developed at the Wellcome Sanger Institute (WSI) Tree of Life Core Laboratory are available on protocols.io ( Howard *et al.*, 2025). The bGalChl1 sample was weighed and triaged to determine the appropriate extraction protocol. Tissue from the muscle was homogenised by cryogenic disruption using the Covaris cryoPREP ^®^ Automated Dry Pulverizer.

HMW DNA was extracted in the WSI Scientific Operations core using the Automated MagAttract v2 protocol. DNA was sheared into an average fragment size of 12–20 kb following the Megaruptor®3 for LI PacBio protocol. Sheared DNA was purified by manual SPRI (solid-phase reversible immobilisation). The concentration of the sheared and purified DNA was assessed using a Nanodrop spectrophotometer and Qubit Fluorometer using the Qubit dsDNA High Sensitivity Assay kit. Fragment size distribution was evaluated by running the sample on the FemtoPulse system. For this sample, the final post-shearing DNA had a Qubit concentration of 12.3 ng/μL and a yield of 3 982.00 ng, with a fragment size of 12.8 kb. The 260/280 spectrophotometric ratio was 1.88, and the 260/230 ratio was 1.84.

RNA was extracted from muscle tissue of bGalChl1 in the Tree of Life Laboratory at the WSI using the RNA Extraction: Automated MagMax™ *mir*Vana protocol. The RNA concentration was assessed using a Nanodrop spectrophotometer and a Qubit Fluorometer using the Qubit RNA Broad-Range Assay kit. Analysis of the integrity of the RNA was done using the Agilent RNA 6000 Pico Kit and Eukaryotic Total RNA assay.

### PacBio HiFi library preparation and sequencing

Library preparation and sequencing were performed at the WSI Scientific Operations core. Libraries were prepared using the SMRTbell Prep Kit 3.0 (Pacific Biosciences, California, USA), following the manufacturer’s instructions. The kit includes reagents for end repair/A-tailing, adapter ligation, post-ligation SMRTbell bead clean-up, and nuclease treatment. Size selection and clean-up were performed using diluted AMPure PB beads (Pacific Biosciences). DNA concentration was quantified using a Qubit Fluorometer v4.0 (ThermoFisher Scientific) and the Qubit 1X dsDNA HS assay kit. Final library fragment size was assessed with the Agilent Femto Pulse Automated Pulsed Field CE Instrument (Agilent Technologies) using the gDNA 55 kb BAC analysis kit.

The sample was sequenced on a Revio instrument (Pacific Biosciences). The prepared library was normalised to 2 nM, and 15 μL was used for making complexes. Primers were annealed and polymerases bound to generate circularised complexes, following the manufacturer’s instructions. Complexes were purified using 1.2X SMRTbell beads, then diluted to the Revio loading concentration (200–300 pM) and spiked with a Revio sequencing internal control. The sample was sequenced on a Revio 25M SMRT cell. The SMRT Link software (Pacific Biosciences), a web-based workflow manager, was used to configure and monitor the run and to carry out primary and secondary data analysis.

### Hi-C

***Sample preparation and crosslinking***

The Hi-C sample was prepared from 20–50 mg of frozen muscle tissue of the bGalChl1 sample using the Arima-HiC v2 kit (Arima Genomics). Following the manufacturer’s instructions, tissue was fixed and DNA crosslinked using TC buffer to a final formaldehyde concentration of 2%. The tissue was homogenised using the Diagnocine Power Masher-II. Crosslinked DNA was digested with a restriction enzyme master mix, biotinylated, and ligated. Clean-up was performed with SPRISelect beads before library preparation. DNA concentration was measured with the Qubit Fluorometer (Thermo Fisher Scientific) and Qubit HS Assay Kit. The biotinylation percentage was estimated using the Arima-HiC v2 QC beads.

***Hi-C library preparation and sequencing***

Biotinylated DNA constructs were fragmented using a Covaris E220 sonicator and size selected to 400–600 bp using SPRISelect beads. DNA was enriched with Arima-HiC v2 kit Enrichment beads. End repair, A-tailing, and adapter ligation were carried out with the NEBNext Ultra II DNA Library Prep Kit (New England Biolabs), following a modified protocol where library preparation occurs while DNA remains bound to the Enrichment beads. Library amplification was performed using KAPA HiFi HotStart mix and a custom Unique Dual Index (UDI) barcode set (Integrated DNA Technologies). Depending on sample concentration and biotinylation percentage determined at the crosslinking stage, libraries were amplified with 10–16 PCR cycles. Post-PCR clean-up was performed with SPRISelect beads. Libraries were quantified using the AccuClear Ultra High Sensitivity dsDNA Standards Assay Kit (Biotium) and a FLUOstar Omega plate reader (BMG Labtech).

Prior to sequencing, libraries were normalised to 10 ng/μL. Normalised libraries were quantified again and equimolar and/or weighted 2.8 nM pools. Pool concentrations were checked using the Agilent 4200 TapeStation (Agilent) with High Sensitivity D500 reagents before sequencing. Sequencing was performed using paired-end 150 bp reads on the Illumina NovaSeq X.

### RNA library preparation and sequencing

Libraries were prepared using the NEBNext ^®^ Ultra™ II Directional RNA Library Prep Kit for Illumina (New England Biolabs), following the manufacturer’s instructions. Poly(A) mRNA in the total RNA solution was isolated using oligo(dT) beads, converted to cDNA, and uniquely indexed; 14 PCR cycles were performed. Libraries were size-selected to produce fragments between 100–300 bp. Libraries were quantified, normalised, pooled to a final concentration of 2.8 nM, and diluted to 150 pM for loading. Sequencing was carried out on the Illumina NovaSeq X to generate 150-bp paired-end reads.

### Genome assembly

Prior to assembly of the PacBio HiFi reads, a database of *k*-mer counts ( *k* = 31) was generated from the filtered reads using FastK. GenomeScope2 ( Ranallo-Benavidez *et al.*, 2020) was used to analyse the *k*-mer frequency distributions, providing estimates of genome size, heterozygosity, and repeat content.

The HiFi reads were assembled using Hifiasm in Hi-C phasing mode ( Cheng *et al.*, 2021; Cheng *et al.*, 2022), producing two haplotypes. Hi-C reads ( Rao *et al.*, 2014) were mapped to the primary contigs using bwa-mem2 ( Vasimuddin *et al.*, 2019). Contigs were further scaffolded with Hi-C data in YaHS ( Zhou *et al.*, 2023), using the --break option for handling potential misassemblies. The scaffolded assemblies were evaluated using Gfastats ( Formenti *et al.*, 2022), BUSCO ( Manni *et al.*, 2021) and MERQURY.FK ( Rhie *et al.*, 2020).

The mitochondrial genome was assembled using MitoHiFi ( Uliano-Silva *et al.*, 2023), which runs MitoFinder ( Allio *et al.*, 2020) and uses these annotations to select the final mitochondrial contig and to ensure the general quality of the sequence.

### Assembly curation

The assembly was decontaminated using the Assembly Screen for Cobionts and Contaminants ( ASCC) pipeline. TreeVal was used to generate the flat files and maps for use in curation. MicroFinder ( Mathers *et al.*, 2025) was used to order scaffolds prior to curation. Manual curation was conducted primarily in PretextView and HiGlass ( Kerpedjiev *et al.*, 2018). Scaffolds were visually inspected and corrected as described by Howe *et al*. (2021). Manual corrections included 7 breaks, 63 joins, and removal of 395 haplotypic duplications. The curation process is documented at https://gitlab.com/wtsi-grit/rapid-curation. PretextSnapshot was used to generate a Hi-C contact map of the final assembly.

### Assembly quality assessment

The Merqury.FK tool ( Rhie *et al.*, 2020) was run in a Singularity container ( Kurtzer *et al.*, 2017) to evaluate *k*-mer completeness and assembly quality for both haplotypes using the *k*-mer databases ( *k* = 31) computed prior to genome assembly. The analysis outputs included assembly QV scores and completeness statistics.

The genome was analysed using the BlobToolKit pipeline, a Nextflow implementation of the earlier Snakemake version ( Challis *et al.*, 2020). The pipeline aligns PacBio reads using minimap2 ( Li, 2018) and SAMtools ( Danecek *et al.*, 2021) to generate coverage tracks. It runs BUSCO ( Manni *et al.*, 2021) using lineages identified from the NCBI Taxonomy ( Schoch *et al.*, 2020). For the three domain-level lineages, BUSCO genes are aligned to the UniProt Reference Proteomes database ( Bateman *et al.*, 2023) using DIAMOND blastp ( Buchfink *et al.*, 2021). The genome is divided into chunks based on the density of BUSCO genes from the closest taxonomic lineage, and each chunk is aligned to the UniProt Reference Proteomes database with DIAMOND blastx. Sequences without hits are chunked using seqtk and aligned to the NT database with blastn ( Altschul *et al.*, 1990). The BlobToolKit suite consolidates all outputs into a blobdir for visualisation. The BlobToolKit pipeline was developed using nf-core tooling ( Ewels *et al.*, 2020) and MultiQC ( Ewels *et al.*, 2016), with containerisation through Docker ( Merkel, 2014) and Singularity ( Kurtzer *et al.*, 2017).

## Genome sequence report

### Sequence data

**Figure 2.**
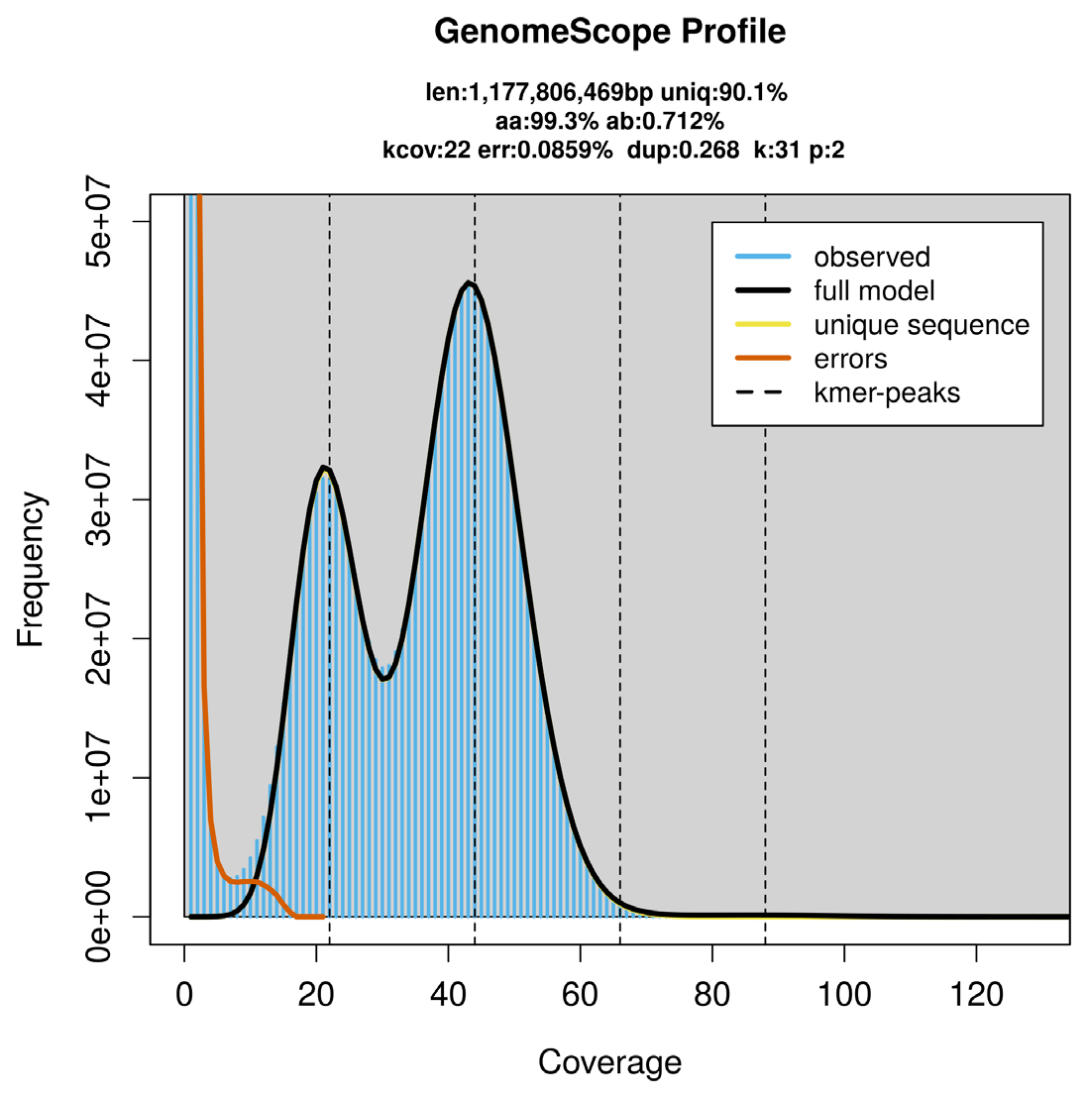
Frequency distribution of *k*-mers generated using GenomeScope2. The plot shows observed and modelled *k*-mer spectra, providing estimates of genome size, heterozygosity, and repeat content based on unassembled sequencing reads.

PacBio sequencing of the *Gallinula chloropus* specimen generated 53.43 Gb (gigabases) from 6.61 million reads, which were used to assemble the genome. GenomeScope2.0 analysis estimated the haploid genome size at 1 177.81 Mb, with a heterozygosity of 0.71% and repeat content of 9.94% ( Figure 2). These estimates guided expectations for the assembly. Based on the estimated genome size, the sequencing data provided approximately 44× coverage. Hi-C sequencing produced 56.12 Gb from 371.67 million reads, which were used to scaffold the assembly. RNA sequencing data were also generated and are available in public sequence repositories. Table 1 summarises the specimen and sequencing details.

**Table 1.** Specimen and sequencing data for BioProject PRJEB74708.

Platform	PacBio HiFi	Hi-C	RNA-seq
**ToLID**	bGalChl1	bGalChl1	bGalChl1
**Specimen ID**	NHMUK014561234	NHMUK014561234	NHMUK014561234
**BioSample (source individual)**	SAMEA113398958	SAMEA113398958	SAMEA113398958
**BioSample (tissue)**	SAMEA114299703	SAMEA114299703	SAMEA114299703
**Tissue**	muscle	muscle	muscle
**Instrument**	Revio	Illumina NovaSeq X	Illumina NovaSeq X
**Run accessions**	ERR12875176	ERR12893025	ERR13493939
**Read count total**	6.61 million	371.67 million	71.64 million
**Base count total**	53.43 Gb	56.12 Gb	10.82 Gb

### Assembly statistics

The genome was assembled into two haplotypes using Hi-C phasing. Haplotype 1 was curated to chromosome level, while haplotype 2 was assembled to scaffold level. The final assembly has a total length of 1 282.39 Mb in 817 scaffolds, with 603 gaps, and a scaffold N50 of 90.34 Mb ( Table 2).

**Table 2.** Genome assembly statistics.

Metric	Haplotype 1	Haplotype 2
**Assembly name**	bGalChl1.hap1.1	bGalChl1.hap2.1
**Assembly accession**	GCA_964237585.1	GCA_964237395.1
**Assembly level**	chromosome	scaffold
**Span (Mb)**	1 282.39	1 208.56
**Number of chromosomes**	38	N/A
**Number of contigs**	1 420	931
**Contig N50**	3.67 Mb	4.02 Mb
**Number of scaffolds**	817	395
**Scaffold N50**	90.34 Mb	87.26 Mb
**Longest scaffold length (Mb)**	215.89	N/A
**Sex chromosomes**	Z	N/A
**Organelles**	Mitochondrion: 17.04 kb	N/A

**Figure 3.**
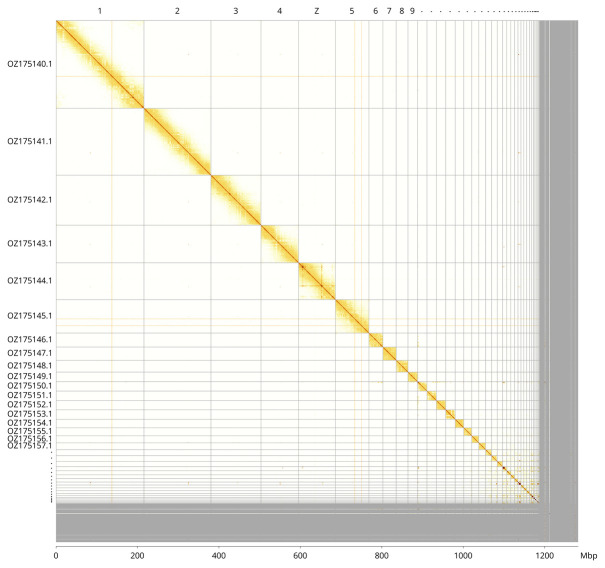
Hi-C contact map of the *Gallinula chloropus* genome assembly. Assembled chromosomes are shown in order of size and labelled along the axes, with a megabase scale shown below. The plot was generated using PretextSnapshot.

Most of the assembly sequence (92.66%) was assigned to 38 chromosomal-level scaffolds, representing 37 autosomes and the Z sex chromosome. These chromosome-level scaffolds, confirmed by Hi-C data, are named according to size ( Figure 3; Table 3).

**Table 3.** Chromosomal pseudomolecules in the haplotype 1 genome assembly of *Gallinula chloropus* bGalChl1.

INSDC accession	Molecule	Length (Mb)	GC%
OZ175140.1	1	215.89	42
OZ175141.1	2	164.19	41.50
OZ175142.1	3	122.94	42
OZ175143.1	4	92.36	42
OZ175145.1	5	82.23	43
OZ175146.1	6	34.21	43.50
OZ175147.1	7	32.66	41.50
OZ175148.1	8	28.86	44.50
OZ175149.1	9	23.46	45
OZ175150.1	10	23.22	46
OZ175151.1	11	23.07	45
OZ175152.1	12	23.04	45
OZ175153.1	13	23.01	44
OZ175154.1	14	21.23	44.50
OZ175155.1	15	19.38	47.50
OZ175156.1	16	17.44	48
OZ175157.1	17	16.81	47.50
OZ175158.1	18	14.42	49.50
OZ175159.1	19	14.13	49
OZ175160.1	20	12.82	48.50
OZ175161.1	21	10.40	49
OZ175162.1	22	10.09	52.50
OZ175163.1	23	8.83	49.50
OZ175164.1	24	8.65	51.50
OZ175165.1	25	7.33	56
OZ175166.1	26	7.23	52.50
OZ175167.1	27	7.02	53.50
OZ175168.1	28	6.91	54
OZ175169.1	29	5.29	55
OZ175170.1	30	5.07	52.50
OZ175171.1	31	3.59	58
OZ175172.1	32	3.57	54
OZ175173.1	33	3	60.50
OZ175174.1	34	2.55	53.50
OZ175175.1	35	2.05	51.50
OZ175176.1	36	0.75	61.50
OZ175177.1	37	0.21	64.50
OZ175144.1	Z	90.34	42

The mitochondrial genome was also assembled. This sequence is included as a contig in the multifasta file of the genome submission and as a standalone record.

**Figure 4.**
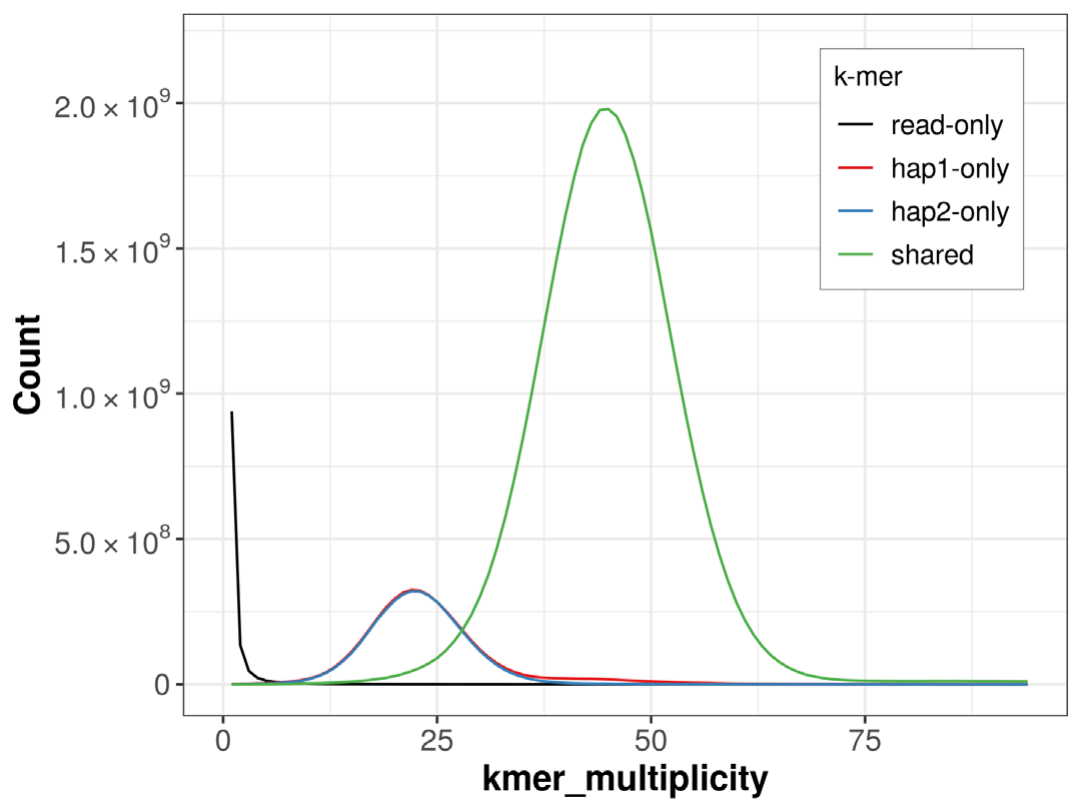
Evaluation of *k*-mer completeness using MerquryFK. This plot illustrates the recovery of *k*-mers from the original read data in the final assemblies. The horizontal axis represents *k*-mer multiplicity, and the vertical axis shows the number of *k*-mers. The black curve represents *k*-mers that appear in the reads but are not assembled. The green curve corresponds to *k*-mers shared by both haplotypes, and the red and blue curves show *k*-mers found only in one of the haplotypes.

For haplotype 1, the estimated QV is 62.5, and for haplotype 2, 63.4. When the two haplotypes are combined, the assembly achieves an estimated QV of 62.9. The *k*-mer completeness is 84.64% for haplotype 1, 83.77% for haplotype 2, and 99.81% for the combined haplotypes ( Figure 4).

**Figure 5.**
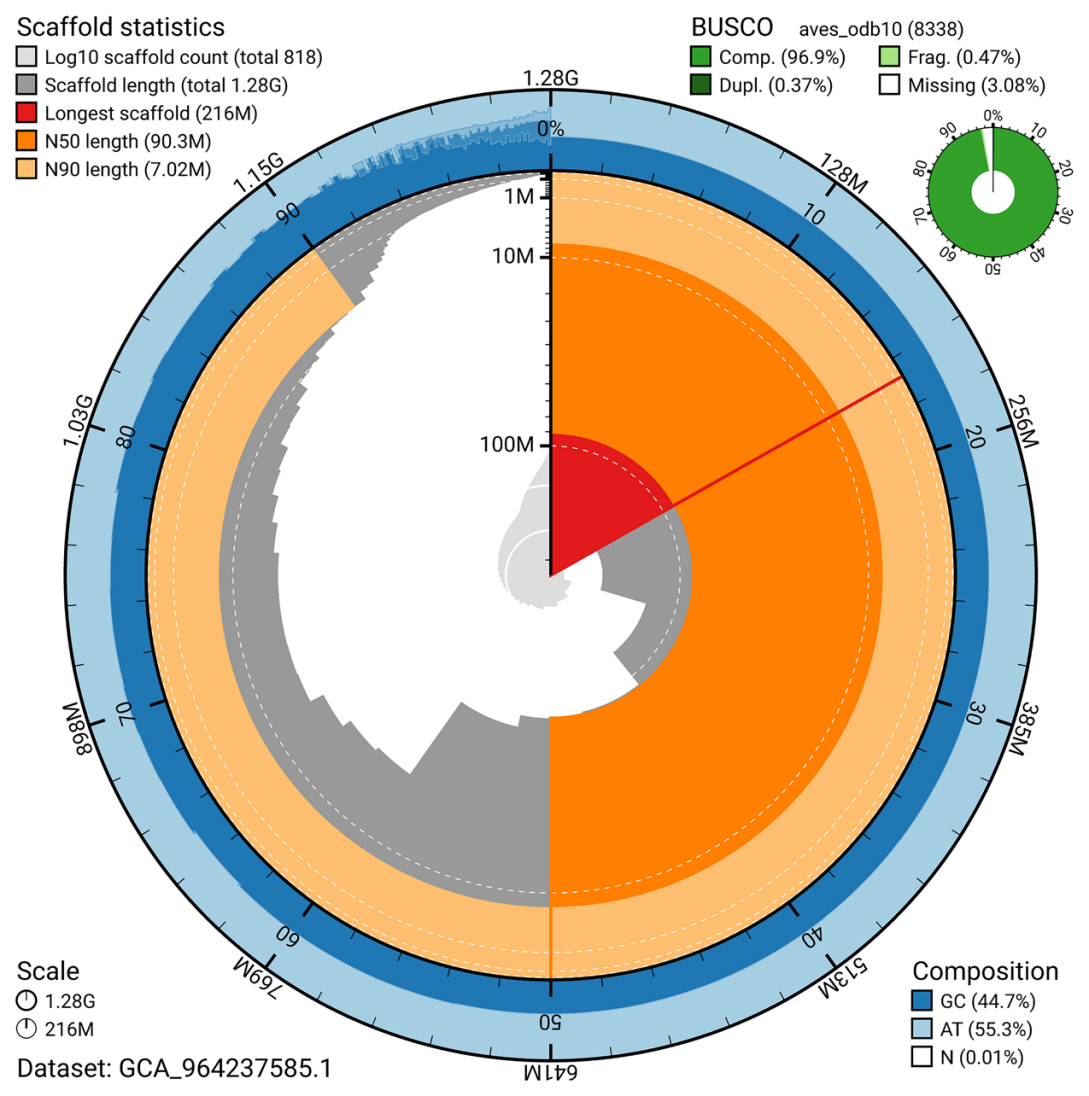
Assembly metrics for bGalChl1.hap1.1. The BlobToolKit snail plot provides an overview of assembly metrics and BUSCO gene completeness. The circumference represents the length of the whole genome sequence, and the main plot is divided into 1 000 bins around the circumference. The outermost blue tracks display the distribution of GC, AT, and N percentages across the bins. Scaffolds are arranged clockwise from longest to shortest and are depicted in dark grey. The longest scaffold is indicated by the red arc, and the deeper orange and pale orange arcs represent the N50 and N90 lengths. A light grey spiral at the centre shows the cumulative scaffold count on a logarithmic scale. A summary of complete, fragmented, duplicated, and missing BUSCO genes in the set is presented at the top right. An interactive version of this figure can be accessed on the BlobToolKit viewer.

**Figure 6.**
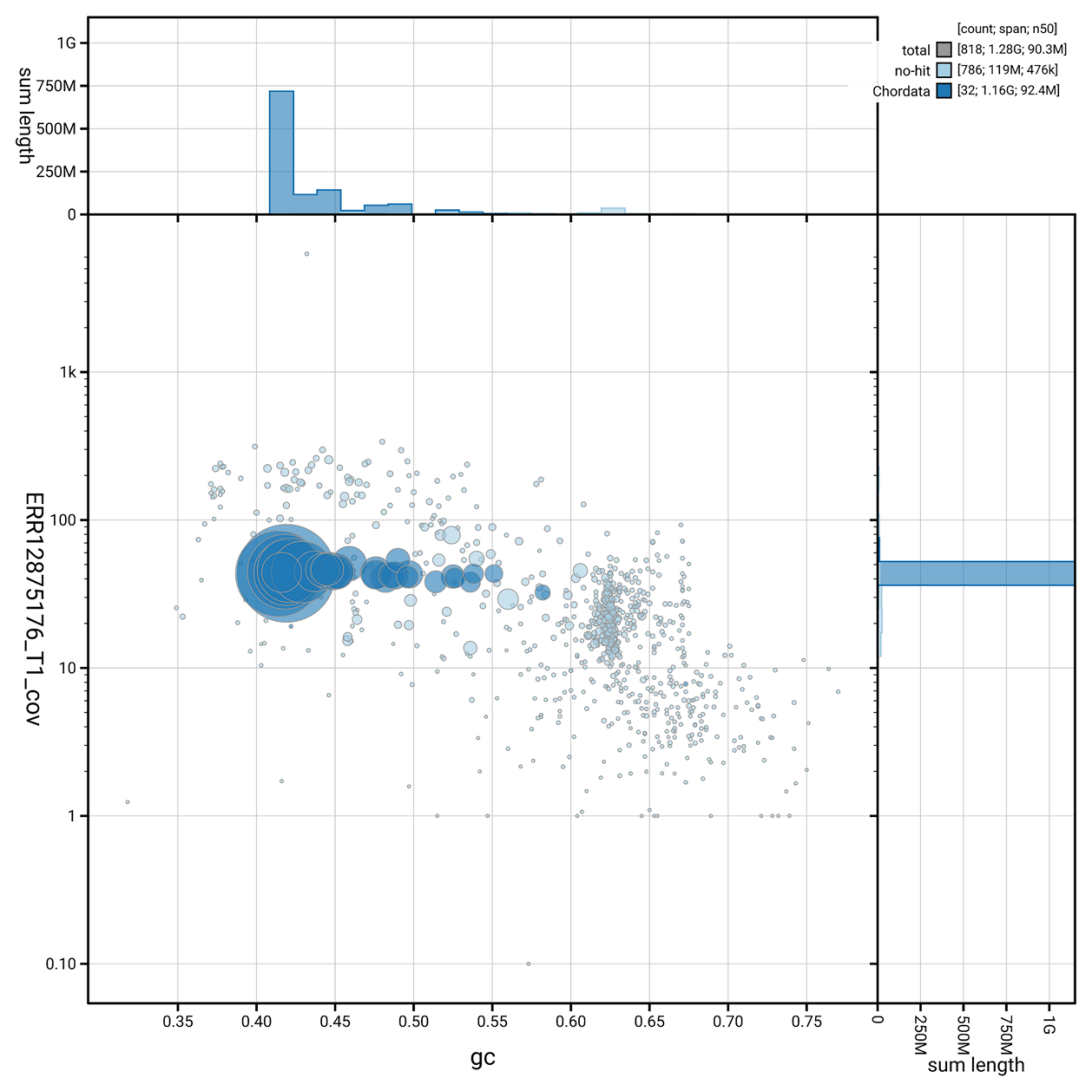
BlobToolKit GC-coverage plot for bGalChl1.hap1.1. Blob plot showing sequence coverage (vertical axis) and GC content (horizontal axis). The circles represent scaffolds, with the size proportional to scaffold length and the colour representing phylum membership. The histograms along the axes display the total length of sequences distributed across different levels of coverage and GC content. An interactive version of this figure is available on the BlobToolKit viewer.

BUSCO analysis using the aves_odb10 reference set ( *n* = 8 338) identified 96.9% of the expected gene set (single = 96.5%, duplicated = 0.4%) for haplotype 1. The snail plot in Figure 5 summarises the scaffold length distribution and other assembly statistics for haplotype 1. The blob plot in Figure 6 shows the distribution of scaffolds by GC proportion and coverage for haplotype 1.

Table 4 lists the assembly metric benchmarks adapted from Rhie *et al.* (2021) the Earth BioGenome Project Report on Assembly Standards September 2024. The EBP metric, calculated for the haplotype 1, is **6.C.Q62**, meeting the recommended reference standard.

**Table 4.** Earth Biogenome Project summary metrics for the *Gallinula chloropus* assembly.

Measure	Value	Benchmark
EBP summary (haplotype 1)	6.C.Q62	6.C.Q40
Contig N50 length	3.67 Mb	≥ 1 Mb
Scaffold N50 length	90.34 Mb	= chromosome N50
Consensus quality (QV)	Haplotype 1: 62.5; haplotype 2: 63.4; combined: 62.9	≥ 40
*k*-mer completeness	Haplotype 1: 84.64%; Haplotype 2: 83.77%; combined: 99.81%	≥ 95%
BUSCO	C:96.9% [S:96.5%; D:0.4%]; F:0.5%; M:2.6%; n:8 338	S > 90%; D < 5%
Percentage of assembly assigned to chromosomes	92.66%	≥ 90%

### Wellcome Sanger Institute – Legal and Governance

The materials that have contributed to this genome note have been supplied by a Darwin Tree of Life Partner. The submission of materials by a Darwin Tree of Life Partner is subject to the **‘Darwin Tree of Life Project Sampling Code of Practice’**, which can be found in full on the Darwin Tree of Life website. By agreeing with and signing up to the Sampling Code of Practice, the Darwin Tree of Life Partner agrees they will meet the legal and ethical requirements and standards set out within this document in respect of all samples acquired for, and supplied to, the Darwin Tree of Life Project. Further, the Wellcome Sanger Institute employs a process whereby due diligence is carried out proportionate to the nature of the materials themselves, and the circumstances under which they have been/are to be collected and provided for use. The purpose of this is to address and mitigate any potential legal and/or ethical implications of receipt and use of the materials as part of the research project, and to ensure that in doing so we align with best practice wherever possible. The overarching areas of consideration are:

•   Ethical review of provenance and sourcing of the material

•   Legality of collection, transfer and use (national and international)

Each transfer of samples is further undertaken according to a Research Collaboration Agreement or Material Transfer Agreement entered into by the Darwin Tree of Life Partner, Genome Research Limited (operating as the Wellcome Sanger Institute), and in some circumstances, other Darwin Tree of Life collaborators.

## Data Availability

European Nucleotide Archive: Gallinula chloropus (common moorhen). Accession number PRJEB74708. The genome sequence is released openly for reuse. The *Gallinula chloropus* genome sequencing initiative is part of the Darwin Tree of Life Project (PRJEB40665), the Sanger Institute Tree of Life Programme (PRJEB43745) and the Vertebrate Genomes Project (PRJNA489243). All raw sequence data and the assembly have been deposited in INSDC databases. The genome will be annotated using available RNA-Seq data and presented through the Ensembl pipeline at the European Bioinformatics Institute. Raw data and assembly accession identifiers are reported in Table 1 and Table 2. Production code used in genome assembly at the WSI Tree of Life is available at https://github.com/sanger-tol. Table 5 lists software versions used in this study.

## Author information

Contributors are listed at the following links:

•   Members of the Natural History Museum Genome Acquisition Lab

•   Members of the Wellcome Sanger Institute Tree of Life Management, Samples and Laboratory team

•   Members of Wellcome Sanger Institute Scientific Operations – Sequencing Operations

•   Members of the Wellcome Sanger Institute Tree of Life Core Informatics team

•   Members of the Tree of Life Core Informatics collective

•   Members of the Darwin Tree of Life Consortium

## References

[ref-1] Allio R Schomaker-Bastos A Romiguier J : MitoFinder: efficient automated large-scale extraction of mitogenomic data in target enrichment phylogenomics. *Mol Ecol Resour.* 2020;20(4):892–905. 10.1111/1755-0998.13160 32243090 PMC7497042

[ref-2] Altschul SF Gish W Miller W : Basic Local Alignment Search Tool. *J Mol Biol.* 1990;215(3):403–410. 10.1016/S0022-2836(05)80360-2 2231712

[ref-3] Amininasab SM Hosseini-Moosavi SM Xu CC : Influence of breeding time, nest size, and egg size on the breeding success of the Common Moorhen *Gallinula chloropus*. *Acta Oecologica.* 2021;113: 103779. 10.1016/j.actao.2021.103779

[ref-4] Anza I Vidal D Feliu J : Differences in the vulnerability of waterbird species to botulism outbreaks in mediterranean wetlands: an assessment of ecological and physiological factors. *Appl Environ Microbiol.* 2016;82(10):3092–99. 10.1128/AEM.00119-16 27016572 PMC4959067

[ref-5] BASC: Sustainable shooting code of practice. 2025. Reference Source

[ref-6] Bateman A Martin MJ Orchard S : UniProt: the Universal Protein Knowledgebase in 2023. *Nucleic Acids Res.* 2023;51(D1):D523–D531. 10.1093/nar/gkac1052 36408920 PMC9825514

[ref-7] Buchfink B Reuter K Drost HG : Sensitive protein alignments at Tree-of-Life scale using DIAMOND. *Nat Methods.* 2021;18(4):366–368. 10.1038/s41592-021-01101-x 33828273 PMC8026399

[ref-8] Challis R Richards E Rajan J : BlobToolKit – interactive quality assessment of genome assemblies. *G3 (Bethesda).* 2020;10(4):1361–1374. 10.1534/g3.119.400908 32071071 PMC7144090

[ref-9] Cheng H Concepcion GT Feng X : Haplotype-resolved *de novo* assembly using phased assembly graphs with hifiasm. *Nat Methods.* 2021;18(2):170–175. 10.1038/s41592-020-01056-5 33526886 PMC7961889

[ref-10] Cheng H Jarvis ED Fedrigo O : Haplotype-resolved assembly of diploid genomes without parental data. *Nat Biotechnol.* 2022;40(9):1332–1335. 10.1038/s41587-022-01261-x 35332338 PMC9464699

[ref-11] Ciach M : Moorhen and little crake feeding on carrion. *Berkut Ukrainian Ornithological Journal.* 2004;13(2):300. Reference Source

[ref-12] Danecek P Bonfield JK Liddle J : Twelve years of SAMtools and BCFtools. *GigaScience.* 2021;10(2): giab008. 10.1093/gigascience/giab008 33590861 PMC7931819

[ref-13] eBird: Common Moorhen *Gallinula chloropus*. 2025. Reference Source

[ref-14] Ewels P Magnusson M Lundin S : MultiQC: summarize analysis results for multiple tools and samples in a single report. *Bioinformatics.* 2016;32(19):3047–3048. 10.1093/bioinformatics/btw354 27312411 PMC5039924

[ref-15] Ewels PA Peltzer A Fillinger S : The nf-core framework for community-curated bioinformatics pipelines. *Nat Biotechnol.* 2020;38(3):276–278. 10.1038/s41587-020-0439-x 32055031

[ref-16] Foreman DW : The breeding ecology of the moorhen, Gallinula chloropus, in an artificially created wetland environment at WWT Llanelli, South Wales. 2001. Reference Source

[ref-17] Formenti G Abueg L Brajuka A : Gfastats: conversion, evaluation and manipulation of genome sequences using assembly graphs. *Bioinformatics.* 2022;38(17):4214–4216. 10.1093/bioinformatics/btac460 35799367 PMC9438950

[ref-18] Gill F Donsker D Rasmussen P : IOC world bird list v15.1, finfoots, flufftails, rails, trumpeters, cranes, Limpkin. 2025. Reference Source

[ref-19] Howard C Denton A Jackson B : On the path to reference genomes for all biodiversity: lessons learned and laboratory protocols created in the Sanger Tree of Life core laboratory over the first 2000 species. *bioRxiv.* 2025. 10.1101/2025.04.11.648334 PMC12548527 41129326

[ref-20] Howe K Chow W Collins J : Significantly improving the quality of genome assemblies through curation. *GigaScience.* 2021;10(1): giaa153. 10.1093/gigascience/giaa153 33420778 PMC7794651

[ref-21] del Hoyo J Elliott A Sargatal J : Handbook of the birds of the world: Volume 3: hoatzin to auks.Barcelona, Spain: Lynx Edicions;1996.

[ref-22] Huxley CR Wood NA : Aspects of the breeding of the moorhen in Britain. *Bird Study.* 1976;23(1):1–10. 10.1080/00063657609476478

[ref-23] IUCN: Common moorhen, *Gallinula chloropus*. 2019. Reference Source

[ref-24] Kerpedjiev P Abdennur N Lekschas F : HiGlass: web-based visual exploration and analysis of genome interaction maps. *Genome Biol.* 2018;19(1): 125. 10.1186/s13059-018-1486-1 30143029 PMC6109259

[ref-25] Kurtzer GM Sochat V Bauer MW : Singularity: scientific containers for mobility of compute. *PLoS One.* 2017;12(5): e0177459. 10.1371/journal.pone.0177459 28494014 PMC5426675

[ref-26] Lardjane-Hamiti A Metna F Boukhemza M : Variation in the diet of Common Moorhen *Gallinula chloropus* (Aves, Rallidae) at Lake Réghaïa, Algeria. *Zool Ecol.* 2015;25(3):227–34. 10.1080/21658005.2015.1046270

[ref-27] Lawniczak MKN Davey RP Rajan J : Specimen and sample metadata standards for biodiversity genomics: a proposal from the Darwin Tree of Life project [version 1; peer review: 2 approved with reservations]. *Wellcome Open Res.* 2022;7:187. 10.12688/wellcomeopenres.17605.1

[ref-28] Li H : Minimap2: pairwise alignment for nucleotide sequences. *Bioinformatics.* 2018;34(18):3094–3100. 10.1093/bioinformatics/bty191 29750242 PMC6137996

[ref-29] López-Perea JJ Laguna C Jiménez-Moreno M : Metals and metalloids in blood and feathers of common moorhens ( *Gallinula chloropus*) from wetlands that receive treated wastewater. *Sci Total Environ.* 2019;646:84–92. 10.1016/j.scitotenv.2018.07.265 30048871

[ref-30] Manni M Berkeley MR Seppey M : BUSCO update: novel and streamlined workflows along with broader and deeper phylogenetic coverage for scoring of eukaryotic, prokaryotic, and viral genomes. *Mol Biol Evol.* 2021;38(10):4647–4654. 10.1093/molbev/msab199 34320186 PMC8476166

[ref-31] Martelli L Fornasiero D Martínez-Lanfranco JA : Exploring the role of wild bird species in the transmission of avian influenza to poultry. *Transbound Emerg Dis.* 2025;25(1): 2288535. 10.1155/tbed/2288535

[ref-32] Mathers TC Paulini M Sotero-Caio CG : MicroFinder: conserved gene-set mapping and assembly ordering for manual curation of bird microchromosomes. *bioRxiv.* 2025. 10.1101/2025.05.09.653066 PMC13192246 41934175

[ref-33] Merkel D : Docker: lightweight Linux containers for consistent development and deployment. *Linux J.* 2014;2014(239): 2. Reference Source

[ref-34] Miller MP Mullins TD Haig SM : Genetic structure, diversity, and interisland dispersal in the endangered Mariana Common Moorhen ( *Gallinula chloropus guami*). *Condor: Ornithological Applications.* 2015;117(4):660–69. 10.1650/CONDOR-15-42.1

[ref-35] Petrie M : Reproductive strategies of male and female moorhens ( *Gallinula chloropus*).In: D. Rubenstein and R. Wrangham (eds), *Ecological Aspects of Social Evolution: Birds and Mammals.*Princeton University Press,1987;43–63. 10.1515/9781400858149.43

[ref-36] Ranallo-Benavidez TR Jaron KS Schatz MC : GenomeScope 2.0 and Smudgeplot for reference-free profiling of polyploid genomes. *Nat Commun.* 2020;11(1): 1432. 10.1038/s41467-020-14998-3 32188846 PMC7080791

[ref-37] Rao SSP Huntley MH Durand NC : A 3D map of the human genome at kilobase resolution reveals principles of chromatin looping. *Cell.* 2014;159(7):1665–1680. 10.1016/j.cell.2014.11.021 25497547 PMC5635824

[ref-38] Rhie A McCarthy SA Fedrigo O : Towards complete and error-free genome assemblies of all vertebrate species. *Nature.* 2021;592(7856):737–746. 10.1038/s41586-021-03451-0 33911273 PMC8081667

[ref-39] Rhie A Walenz BP Koren S : Merqury: reference-free quality, completeness, and phasing assessment for genome assemblies. *Genome Biol.* 2020;21(1): 245. 10.1186/s13059-020-02134-9 32928274 PMC7488777

[ref-40] Ruan L Xu W Han Y : Gene flow from multiple sources maintains high genetic diversity and stable population history of common Moorhen *Gallinula chloropus* in China. *Ibis.* 2018;160(4):855–69. 10.1111/ibi.12579

[ref-41] Schoch CL Ciufo S Domrachev M : NCBI taxonomy: a comprehensive update on curation, resources and tools. *Database (Oxford).* 2020;2020:baaa062. 10.1093/database/baaa062 32761142 PMC7408187

[ref-42] Thomas GJ : Autumn and winter feeding ecology of waterfowl at the Ouse Washes, England. *J Zool.* 1982;197(1):131–172. 10.1111/jzo.1982.197.1.131

[ref-43] Uliano-Silva M Ferreira JGRN Krasheninnikova K : MitoHiFi: a python pipeline for mitochondrial genome assembly from PacBio high fidelity reads. *BMC Bioinformatics.* 2023;24(1): 288. 10.1186/s12859-023-05385-y 37464285 PMC10354987

[ref-44] Van Duyse E Galbusera P Schenck T : Estimating isolation and genetic differentiation in two Belgian populations of moorhens *Gallinula chloropus.* by using minisatellite and microsatellite markers. *Belg J Zool.* 1999;129:113–24. Reference Source

[ref-45] Vasimuddin M Misra S Li H : Efficient architecture-aware acceleration of BWA-MEM for multicore systems.In: *2019 IEEE International Parallel and Distributed Processing Symposium (IPDPS).*IEEE,2019;314–324. 10.1109/IPDPS.2019.00041

[ref-46] Zamani-Ahmadmahmoodi R Esmaili-Sari A Ghasempouri SM : Mercury in wetland birds of Iran and Iraq: contrasting resident Moorhen, *Gallinula chloropus* and migratory common teal, *Anas crecca,* life strategies. *Bull Environ Contam Toxicol.* 2009;82(4):450–53. 10.1007/s00128-009-9637-4 19142558

[ref-47] Zhou C McCarthy SA Durbin R : YaHS: Yet another Hi-C Scaffolding tool. *Bioinformatics.* 2023;39(1): btac808. 10.1093/bioinformatics/btac808 36525368 PMC9848053

