# Supplementary material for: The genome sequence of the Common Moorhen, Gallinula chloropus (Linnaeus, 1758) (Gruiformes: Rallidae)
Source: Wellcome Open Res. 2025 Oct 3;10:539. [Version 1] doi: 10.12688/wellcomeopenres.24858.1 (PMC12699211; doi:10.12688/wellcomeopenres.24858.1)
Supplement: Peer review history

# Reviewer report: Reviewer response for version 1

**DOI:** 10.21956/wellcomeopenres.27382.r140070

**Published:** 2025-12-12

**Authors:** Stephan Koblmüller

**Affiliations:** 1 University of Graz, Graz, Graz, Austria

In this data note, Humby *et al*. present the genome assembly of the Common Moorhen, a rallid species widely distributed across many parts of Europe, Africa and Asia. I particularly like the background section, which gives an informative introduction to the study species. The data are clear, and the methods are well described. I have no objections to any of the data presented or the results. As far as I can tell, the datasets are deposited in publicly accessible repositories.

Are sufficient details of methods and materials provided to allow replication by others?

Yes

Is the rationale for creating the dataset(s) clearly described?

Yes

Are the datasets clearly presented in a useable and accessible format?

Yes

Are the protocols appropriate and is the work technically sound?

Yes

Reviewer Expertise:

phylogenetics/-genomics, population genetics, phylogeography

I confirm that I have read this submission and believe that I have an appropriate level of expertise to confirm that it is of an acceptable scientific standard.

# Reviewer report: Reviewer response for version 1

**DOI:** 10.21956/wellcomeopenres.27382.r140069

**Published:** 2025-12-11

**Authors:** Tsuneyuki Tatsuke

**Affiliations:** 1 National Agriculture and Food Research Organization, Tsukuba, Ibaraki, Japan

The authors successfully sequenced the genomic DNA of the Common Moorhen, *Gallinula chloropus*, with high accuracy, primarily using the PacBio Revio system, which resulted in the construction of a novel reference genome. This study provides detailed documentation of the collection time, location, and preservation status of the sequenced individual, and the experimental methodology is thoroughly described. This reference genomic sequence and associated data are publicly available online and are expected to serve as a valuable foundation for future research.

Are sufficient details of methods and materials provided to allow replication by others?

Yes

Is the rationale for creating the dataset(s) clearly described?

Yes

Are the datasets clearly presented in a useable and accessible format?

Yes

Are the protocols appropriate and is the work technically sound?

Yes

Reviewer Expertise:

Molecular Biology

I confirm that I have read this submission and believe that I have an appropriate level of expertise to confirm that it is of an acceptable scientific standard.

# Reviewer report: Reviewer response for version 1

**DOI:** 10.21956/wellcomeopenres.27382.r135377

**Published:** 2025-10-14

**Authors:** Xuejing Wang

**Affiliations:** 1 Copenhagen University, Copenha, Denmark

This paper made a clear and thorough description of the newly assembled reference genome for the common moorhen. Overall it is a very good work. The methods of sampling, sequencing and assembling are sound. The quality of the assembly is very high. The data of the reference genome is available online.

 I only have a small question about RNA sequencing, or rather the specimen. Usually it is better to take RNA samples shortly after the death of the animal. Nevertheless, looking by the photo of the bird, it seemed very fresh, and the sequencing quality looks high. Maybe it is good to have a short description, like one sentence or a phrase, about the condition of the specimen?

Are sufficient details of methods and materials provided to allow replication by others?

Yes

Is the rationale for creating the dataset(s) clearly described?

Yes

Are the datasets clearly presented in a useable and accessible format?

Yes

Are the protocols appropriate and is the work technically sound?

Yes

Reviewer Expertise:

Population genetics and conservation genomics

I confirm that I have read this submission and believe that I have an appropriate level of expertise to confirm that it is of an acceptable scientific standard.
